# A mixed method examination: how stigma experienced by autistic adults relates to metrics of social identity and social functioning

**DOI:** 10.3389/fpsyt.2023.1243618

**Published:** 2023-10-26

**Authors:** Alex Marion, Karrah Bowman, Gina Thomas, Ashley J. Harrison

**Affiliations:** ^1^Medical College of Georgia, Augusta University, Augusta, GA, United States; ^2^Department of Educational Psychology, University of Georgia, Athens, GA, United States

**Keywords:** stigma, autism, social identity, self-esteem, self-efficacy, social satisfaction, neurodiversity

## Abstract

A recent meta-analysis reveals almost half of autistic individuals experience some form of victimization in their lifetime, including bullying and other forms of stigma. Research among caregivers of autistic individuals demonstrates that stigma can have a long-lasting impact on other aspects of a social identity, such as self-esteem, but less research has specifically examined this among autistic adults themselves, in spite of research suggesting these are likely constructs that contribute to the internalization of stigma and subsequent mental health consequences. The current study used a mixed method approach to assess the relation between stigma and several components of social identity and social functioning. More specifically, among 45 autistic young adults, three dimensions of self-reported stigma (discrimination, disclosure, and positive aspects) were examined in relation to self-esteem, self-efficacy, social satisfaction and adaptive social functioning. Quantitative analyses revealed higher reported discriminative and disclosure stigma were significantly associated with lower self-efficacy. Increased experience with all types of stigma were associated with lower social satisfaction. Greater reported disclosure stigma was also associated with lower self-esteem. Qualitative interviewing among eight autistic young adults helped to better understand the nature of stigma and the impact of these experiences. Thematic analysis of the qualitative data revealed that all of the participants experienced stigma in the form of exclusion or isolation and that a majority also experienced verbal bullying. Many of the negative interactions came from educators, peers, and family members. Most participants indicated that these stigmatizing interactions directly contributed to decreased social satisfaction, diminished self-efficacy, and lowered self-esteem. A greater understanding of the negative consequences of stigma can inform efforts to increase awareness and acceptance of autism.

## 1. Introduction

### 1.1. Neurodiversity movement and stigma

The autistic community has been at the frontline of the neurodiversity movement, which emphasizes neurological differences as innate, attributable to the person, and simply part of a continuum of human diversity (1). The neurodiversity framework has the potential to reduce stigma [social exclusion due to differences that are perceived to deviate from societal norms; (2)], by explaining that the differences between neurotypical and neurodiverse individuals are due to biology and outside of one’s control (3). This is particularly important to the autism community as these individuals are at heightened risk of experiencing stigma likely because of differences in social functioning and noticeable stereotyped behavior, paired with typical physical appearance (4, 5). Thus, autism allies support this movement to help both decrease the stigmatization of autism and increase the wellbeing of the autistic individuals through increased community acceptance and awareness that the concept of “typical” is driven by societal standards (1, 5).

As a result of the neurodiversity movement, more recent research has focused on the experience of stigma from the perspective of autistic individuals (6). This research reveals that in spite of greater societal acceptance of individual differences through the adoption of neurodiversity framework (7), many autistic adults continue to encounter stigma. Recent meta analyses revealed that 44 to 67% of autistic adults report experiencing stigma (8, 9). Several contemporary literature reviews reveal that autistic individuals continue to experience stigma in many different forms and from various sources (5, 10). However, much of the research included in these recent literature reviews and meta-analyses include data over a broad period of time, which in many cases can be over a decade old (5, 8, 10). Thus, more research needs to examine the current perspective of autistic individuals to better understand the contexts in which stigmatization continues to occur and the extent of consequences of experienced stigma (5, 10). To continue to make gains in stigma reduction, we need to better understand the types of stigma autistic adults continue to experience over time and the primary sources. As we continue working toward shifting societal attitudes, understanding the widespread impact that stigma has on neurodiverse individuals can also help inform approaches to mitigate some of the negative consequences (11).

### 1.2. Contextualizing experienced stigma

The hope is that an adoption of a neurodiversity framework will help to reduce stigma experienced by autistic individuals; however, to gauge success with shifting societal attitudes, research needs to focus on frequently assessing the continued scope of experienced stigma. Further, because research documents that parents might underestimate the extent their children experience stigma (12), it is crucial to specifically document the lived experience from the perspective of autistic individuals.

#### 1.2.1. Perpetrators of stigma

A systematic review reported the majority of experienced stigma over the last decade has originated from peers, teachers, employers/co-workers, and family members (10). Many studies document that autistic individuals feel misunderstood, rejected, and/or excluded by peers. They also found teachers and employers often make false assumptions about an individual’s abilities and needs. Similarly, a metanalysis focused on experiences of stigma in the form of bullying among autistic students and found these instances occurred most frequently in the inclusive classroom setting (8).

In addition to the individual level, autistic people report being impacted by stigma at a group or societal level. For example, media representations of autism are frequently negative, depicting individuals as dangerous and/or unloved (13). Additionally, one study suggested the news coverage of autism may be unintentionally stigmatizing autistic individuals by including stigmatizing cues, such as describing psychiatric symptoms or social skills deficits which distinguish autistic from allistic children, in more than two-thirds of media coverage (14). This same narrative extends into autistic adults, in which dehumanizing language continues to appear (15, 16).

#### 1.2.2. Types of experienced stigma

Both qualitative and quantitative research provides evidence that autistic individuals have historically experienced stigma in alignment with most of the primary modalities of stigma defined in the literature [i.e., labeling, stereotyping, separation, status loss, discrimination and misuse of power; (17)]. For example, qualitative interviews documented that autistic individuals experienced stereotyping, exclusion, and discrimination (18). A systematic review examining the impact of stigma experienced by autistic individuals similarly found evidence for varying types of experienced stigma, including stereotyping, bullying, and judgment (10).

The vast majority of the literature focuses on bullying, which is discussed by most as “ongoing and deliberate misuse of power in relationships through repeated verbal, physical, and/or social behavior that intends to cause physical, social, and/or psychological harm” (8). This type of stigma can manifest as exclusion (19), but is often physical in nature (20). The quantitative research is clear that bullying occurs more readily among autistic individuals compared to those with other disabilities and compared to neurotypical peers (19). One study revealed that 36% of autistic individuals had some lifetime experience of bullying (19) and another documented that as many as 14% had experienced cyber bullying (12); however, a more recent metanalysis revealed pooled prevalence rates of bullying closer to 67% among autistic individuals (8). Additional qualitative research emphasized the pervasive nature of experienced stigma among autistic adults (18). These different types of methodological approaches help to reveal the importance of mixed methods in understanding more about the types of stigma that continue to occur and from what perpetrators. A greater understanding of what types of stigma persist can inform targeted approaches to diminish the negative impact of stigma still experienced by so many autistic adults.

### 1.3. Consequences of experienced stigma

Although the impact of experienced stigma is underrepresented in the literature for autistic adults compared to adults with other developmental disorders, a growing body of literature has also begun to document the myriad of negative consequences among autistic individuals. Growing literature has documented a relation between stigma and mental health in autism. For example, almost half of adults with Asperger’s syndrome had long term sequelae from prior bullying, such as increased levels of anxiety (8). Additionally, there is a known relationship between higher levels of experienced bullying and victims’ increased rates of depression, anxiety, suicidality, and other broad internalizing symptoms (12, 21). An illuminating recent study examined the time ordered relation between bullying and mental health among autistic adolescents and documented that bullying predicted internalizing mental health concerns one year later (22). Less is known about how these stigma experiences convey risk for mental health.

#### 1.3.1. Stigma and social identity consequences

A systematic review examining the impact of stigma experienced by autistic individuals found evidence for a host of more nuanced negative outcomes including internalization of stigma, low self-esteem, negative self-labeling, and concealing their diagnosis (10). These more subtle negative consequences likely contribute to later negative mental health outcomes. Theory suggests that experienced stigma is internalized and converted into self-stigma, where autistic individuals begin to view themselves in a negative light as a result of their negative experiences with others (5, 23). Research documents that a meaningful number of autistic individuals experience this self-stigma [e.g., (23)]. In this process of internalizing stigma, autistic individuals then start to view themselves negatively, which results in increased experiences of shame and fear (5, 10). In alignment with the “Why Try? Effect” (24), among autistic individuals, this internalized stigma is thought to result in negative shifts in social identity (23). These impacted factors of social identity include self-esteem and self-efficacy. Self-esteem is defined as how much someone likes themself and is related to self-respect, worthiness, and adequacy (24, 25). Self-efficacy is how capable one believes themself to be of successfully accomplishing tasks, and social self-efficacy refers specifically to the completion of social tasks or interactions (26).

In support of this theory, a broader review revealed that internalized or self-stigma related to self-esteem and self-efficacy (27). Although not explicitly studied among autistic adults, self-esteem is documented as a mechanism by which stigma contributes to negative mental health outcomes among reviews examining the implications of experienced stigma among families of individuals with developmental delays and autism (28–30). Other aspects of one’s identity, such as self-efficacy, have not been similarly examined but are also a likely additional mechanism.

##### 1.3.1.1. Self-esteem and stigma

Although not directly testing the relation between stigma and self-esteem, a systematic review revealed a relation between self-esteem and social support and loneliness, two experiences related to stigma (5). Relatedly in qualitative research, caregivers of autistic children report that experienced affiliative stigma is directly related to self-esteem and that self-esteem mediated the relation between stigma and negative mental health consequences (31). The only known study to directly quantitatively test the link between stigma and self-esteem among autistic adults did not find significant esteem differences among groups that did and did not experience bullying but this is a topic that remains under-investigated (32).

##### 1.3.1.2. Social self-efficacy and stigma

Although even less well examined in the literature, parents of autistic children report that experiencing stigma led them to feel embarrassed and feel less confident in their parenting (33). A later review identified parent confidence as a potential moderator between experienced stigma and parental mental health challenges (34). Only one known study specifically links self-efficacy related to socialization and stigma among autistic adults. In this study, a large majority of the autistic sample endorsed the item, “I can’t contribute anything to society because I have autism,” revealing signs of low social self-efficacy among autistic participants experiencing internalized stigma (35). In spite of these connections drawn between stigma and self-efficacy, no known research has directly examined the relation between these constructs among autistic adults using comprehensive assessments. Examining how stigma relates to social identity among autistic adults provides greater evidence for the importance of stigma reduction and provides insight into the process of how stigma internalization likely happens.

#### 1.3.2. Stigma and social functioning

Social functioning is a broad concept comprised of multiple factors, including social satisfaction and adaptive social skills. Social satisfaction is often assessed by examining constructs such as loneliness, social adequacy, and peer relations/status (36). Examining how personally satisfied an individual is with their social interactions helps to understand one’s own perception of social success (35). Measures of adaptive functioning help to examine social success from a more objective perspective through a comparison of population norms (37, 38). Adaptive behaviors are real-life skills one performs independently to succeed, and include social adaptive skills or practical behaviors that help an individual socialize in society [e.g., understanding social nuances; (37, 38)]. Socialization was found to be the most impaired adaptive domain among autistic participants (39).

##### 1.3.2.1. Social satisfaction and stigma

In relation to stigma, most studies measure subconstructs of social satisfaction, such as loneliness or feelings of isolation [i.e., (36)]. Related to this, autistic participants in a qualitative study reported being outcasted by society due to their differences (18). Participants in this study also revealed that the internalization of this experienced stigma resulted in social isolation as a result of pressure to conform and subsequent avoidance of social situations to prevent judgment from others. An additional study demonstrated that experienced discrimination by autistic individuals resulted in an expectation of later rejection that likely renders an individual to feel more uncomfortable in social situations (40). All of these negative social experiences revealed through qualitative inquiry align with measurement items designed to quantify loneliness/social dissatisfaction (36).

One quantitative study examined the experiences of loneliness and bullying among autistic college students and found that many experienced bullying and reported limited social satisfaction [e.g., feelings of isolation, feeling left out and limited companionship; (19)]. Of note, this study did not examine a relation between these two constructs and both were measured with a limited number of items. Although these identified feelings of loneliness and isolation that arise as a result of stigmatization are likely to lead to low social satisfaction, this relation has not been specifically examined.

##### 1.3.2.2. Adaptive social functioning and stigma

Autistic individuals’ social adaptive functioning has served as a predictor of stigma in past research (33) and emerged as a meaningful predictor of bullying in a metanalysis (8). Yet, impaired social adaptive functioning might also be an outcome of experienced stigma. Autistic individuals are already at an increased risk for developing a co-occurring disorder, such as anxiety (41). Added social stress (e.g., bullying, stigma) can exacerbate or elicit internalizing problems for autistic individuals. Internalizing problems, such as social anxiety and social withdrawal, have been reported as outcomes in bully victims but also might lead to increased social challenges (42). A link between social anxiety and social self-esteem also suggests that higher levels of social fear, avoidance, and physiological reaction are associated with negative attitudes regarding themselves in social situations (43). Given these associations, this study aims to further examine the relationship between stigma and adaptive social functioning in autistic young adults.

### 1.4. Current study

Despite the progress made in increasing acceptance through the neurodiversity movement, autistic individuals still experience stigma; however, the extent of these experiences in more recent years are not fully documented as many of the published reviews and meta-analyses reflect experiences over a wider or dated time period. As such, using a mixed method approach, the current study documented a more recent perspective of the stigmatizing experiences experienced by autistic adults, including the types of stigma this population continues to endure and from what sources. This study also aimed to extend the research by examining in-depth potential social consequences of experienced stigma.

The quantitative component of this study measured the following:

(1) how several aspects of perceived/experienced stigma (discrimination, disclosure, and positive aspects of stigma) relate to social self-efficacy and self-esteem and
(2) how experienced stigma correlated with measures of social functioning, including self-reported adaptive social skills and social satisfaction.

Qualitative interviews were also conducted with autistic adults, to add more depth to the understanding of the extent and context of experienced stigma, as well as the perception of how stigma relates to social identity. Qualitative data allowed for:

(3) documentation of types of stigma experiences that autistic adults continue to experience and the reported sources of this stigma (44), and
(4) a better understanding of the specific ways in which stigma relates to social consequences from the perspective of autistic adults.

## 2. Materials and methods

### 2.1. Procedure

This study used a mixed-method complimentary design involving an initial quantitative component with a qualitative follow-up (45). A sequential sampling design allowed the researchers to gain a general understanding of the topic before following-up with a deeper exploration of the participants’ experiences (46). This study was approved by the university’s Institutional Review Board Committee. Autistic individuals were recruited for the study through various listservs (e.g., university disability resource centers nationwide, state Autism organizations, etc.) and other online advertisements. Fliers were handed out at autism-related events (e.g., conferences, walks, social skills groups), posted on campus buildings, and distributed to therapist offices in the local area.

### 2.2. Participants

The quantitative study included 45 autistic adults (23 males, 21 females, 1 gender not reported) from the United States. Initial screener questions required the participants to self-report if they had both a confirmed diagnosis of autism and were 18 years or older. The individuals’ ages ranged from 18 to 58 years old (*M* = 25.12, SD = 9.50). Twenty-nine adults were enrolled in postsecondary education and 22 held jobs at the time of survey completion. Thirty-three percent of participants lived at home with family members, 33.3% lived independently, and 26.7% lived on campus in university housing. Race and ethnicity was inadvertently not collected as part of the quantitative data collection.

After collecting the battery of self-report surveys, participants were offered the opportunity to participate in an interview. Eight individuals participated in the qualitative follow-up study. Sampling stopped after thematic saturation was reached across interviews (47). Participants were between the ages of 19 and 40 (*M* = 25.13, SD = 8.06), primarily White (75%), and Non-Hispanic or Latino (88%). All participants had some college completed, with most individuals currently completing an undergraduate degree during the time of the interviews.

### 2.3. Data collection

#### 2.3.1. Quantitative questionnaires

Participants completed five rating scales in addition to some demographic questions regarding their academic standing (e.g., graduation year, major) and living situation. Participants were given the option to complete the questionnaires in person or online. The rating scales took approximately 15 to 30 minutes to complete.

##### 2.3.1.1. Stigma Scale

The Stigma Scale (48) is a 28-item measure that assesses perceived and experienced stigma in individuals with mental health disorders. In this study, the phrase “mental health problems” was replaced with “autism spectrum disorder” wherever it appeared in the measure. Autistic participants rated their perceived or experienced stigma on a 5-point Likert scale (0 = strongly disagree to 4 = strongly agree) in the following sub-scales: discrimination (12 items; Cronbach’s α = 0.87), disclosure (11 items; Cronbach’s α = 0.75), and positive aspects (5 items; Cronbach’s α = 0.79). The discrimination subscale assessed more overt types of experienced stigma, such as experienced hostility from others or losing opportunities due to others’ biases. The disclosure subscale assessed negative experiences with disclosing an autism diagnosis or fear surrounding this process. The positive aspects examined any positive experiences as a result of having an autism diagnosis. For all subscales, higher numbers were associated with greater experienced stigma. This scale has high reported psychometric support (internal consistency α = 0.87) (48). In the current study the internal consistency was similarly solid for the total stigma score (Cronbach’s α = 0.85), as well as for the individual subscales (see above).

##### 2.3.1.2. Adapted rosenberg self-esteem scale

The Adapted Rosenberg Self-Esteem Scale (49) is a six item measure that assesses an individual’s self-esteem and overall feelings of self-worth (25) using a 5-point Likert scale (1 = never true to 5 = always true). A higher score on this measure indicates greater self-esteem and feelings of self-worth. The adapted version of the scale was used due to its simplified wording and past use in the mental health context (49). This is one of the most widely used measures of self-esteem (50), with a excellent demonstration of psychometric support [i.e., (51, 52)]. In the current study, good internal consistency was reported (Cronbach’s α = 0.82).

##### 2.3.1.3. Social self-efficacy subscale

The Social Self-Efficacy Subscale consists of six items derived from the Self-Efficacy Scale identified as a unique factor (53). This subscale is a self-report measure of one’s social competence and the perception of success with completing tasks (54). The measure uses a 5-point Likert scale (1 = strongly disagree to 5 = strongly agree) with higher scores indicating greater self-efficacy. Previous studies demonstrate strong psychometric support for this measure (53). The internal consistency calculated for this sample (Cronbach’s α = 0.65), although considered in the low range by some, is considered in the acceptable range according to multiple psychometric experts for a psychological measure used in research [see (55) for a review].

##### 2.3.1.4. Social satisfaction measure

The social satisfaction measure is a compilation of the social distress and companionship sections of the NIH Toolbox Social Relationships subdomain that assesses how fulfilling individuals find their relationships (56). Previous studies have established solid psychometric support for the measure (57). The measure consists of 22 items that factors onto four scales presented in the following order: friendship (5 items; Cronbach’s α = 0.86), loneliness (7 items; Cronbach’s α = 0.95), perceived rejection (5 items; Cronbach’s α = 0.91), and perceived hostility (5 items; Cronbach’s α = 0.91). The measure uses a 5-point Likert scale (1 = never to 5 = always), with a higher score indicating less social satisfaction.

##### 2.3.1.5. ABAS-II

A widely used adaptive functioning measure, the Adaptive Behavior Assessment System (ABAS), assesses three constructs of adaptive behaviors: conceptual, social, and practical (58). The Social Domain of the Adaptive Behavior Assessment, Second Edition consists of 23 items and was used to measure participants’ perceptions of social skills that help them function in daily living (59). The instructions specify that participants rate how often they perform the various social behaviors independently on a four-point scale (0 = not able to 3 = always). A social composite score was calculated and higher scores on the composite reflect more adaptive behavior skills based on participants’ self perception. Prior studies have demonstrated high internal consistency for the Social domain (60). Similarly, high internal consistency for the Social domain was reported in the current study (Cronbach’s α = 0.96). In this sample, participant standard scores fell in the Extremely low range (0.4th percentile) indicating this sample had notable challenges with adaptive functioning compared to same aged peers.

#### 2.3.2. Qualitative interview

Participants’ met one-on-one with a researcher to complete a semi-structured interview that lasted between 45 minutes and 1 hour. An interview guide was created to establish consistency across interviews and to facilitate discussion with the participant. The guide included questions and prompts that related to experienced stigma and factors impacted by the stigma experienced (i.e., self-esteem, self-efficacy, and social satisfaction). Examples of questions asked include, “Can you tell me a time when you were treated unfairly?” and “When have the actions or words of others made you feel as if you can/can’t interact well with people?”

Questions were derived from different published measurement approaches from both the qualitative and quantitative literature. Specifically, the interview included questions assessing experiences and feelings about receiving a diagnosis aligned with qualitative research examining similar questions (61, 62). Broader stigma questions were derived from the Discrimination and Stigma Scale [DISC; (63)], as well as The Stigma Scale (48). The inquiry about social satisfaction aligned with a qualitative interview assessing social experiences among autistic adults (56). Social identity theory was the framework to guide the second part of the qualitative interview. More specifically, the interview focused on two aspects of social identity theoretically impacted by the internalization of stigma: self-esteem and self-efficacy (23). Interview questions assessing self-esteem were derived from both a qualitative interview (61) and from the Rosenberg Self-Esteem Scale (49) and the self-efficacy conversation was guided by items on the Self-efficacy Scale (53).

This study was conducted by researchers who identify as non-autistic. As neurotypical researchers, we acknowledge our privilege in society and recognize the contrast between our experiences and the participants’ experiences. Throughout the research process, we reflected on how our status in society could influence the development of interview questions, connection with participants, and interpretation of responses. Alignment with the neurodiversity mindset and a thorough knowledge of the autism stigma literature was used as a guiding tool throughout this study.

#### 2.3.3. Analysis plan

All statistical analyses were conducted using SPSS software version 26. The statistical significance for the analyses were set at *p* = 0.05. Using two-tailed bivariate correlational analyses, we examined how three types of stigma were related to a range of social variables. More specifically, the three types of stigma (disclosure stigma, discriminative stigma, and positive aspects of autism) were included in all of the correlational analyses. We first examined how stigma was related to several components of social identity, including self-efficacy and self-esteem. Next, we examined the relation between stigma and social outcomes, including the four subdomains of social satisfaction (i.e., friendship, loneliness, rejection, and hostility) and the ABAS social adaptive functioning subdomain.

## 3. Results

### 3.1. Quantitative

#### 3.1.1. Preliminary analyses

Both the stigma measure and all measures of social functioning demonstrated a normal distribution. For the quantitative measures, means and standard deviations for the current sample are reported in Table 1. This table also includes published means to allow for contextualization of the current data within the broader literature.

**TABLE 1:** Descriptive statistics of all quantitative measures.

Variables	Minimum	Maximum	Mean (SD)	Other published means (References)
**Stigma**				
Discrimination	2	44	22.02 (10.27)	29.1 (48)
Disclosure	3	41	20.67 (8.23)	24.7 (48)
Positive Aspects	0	18	6.96 (4.65)	8.8 (48)
Stigma Total	16	95	49.64 (17.00)	62.6 (48)
**Social satisfaction**				
Friendship	5	25	14.67 (5.37)	26.53 (57)
Loneliness	7	35	19.93 (7.88)	12.02 (57)
Rejection	5	25	11.02 (4.52)	16.93 (57)
Hostility	5	25	11.69 (4.81)	16.87 (57)
Self-esteem	11	30	21.80 (4.83)	23.44 (49)
Social adaptive functioning	0	13	4.97 (3.08)	9.9 (75)
Social self-efficacy	6	26	16.98 (4.36)	21.20 (75)

#### 3.1.2. Correlation analyses

Table 2 presents the correlations between the stigma types and all social identity variables. For the social identity variables, analyses revealed individuals with lower reported self-efficacy had significantly higher reported discriminative and disclosure stigma (all *p*’s < or equal to 0.05). However, expressions of positive aspects of stigma were not significantly related to higher self-efficacy (*p* = 0.19). Additionally, lower self-esteem was correlated with greater reported disclosure stigma (*p* < 0.5) and positive aspects of stigma (*p* < 0.5), but it was not significantly associated with discriminative stigma (*p* = 0.37).

**TABLE 2:** Correlations between stigma and all social variables.

Variable	N	1	2	3
**Stigma**				
1. Discrimination	45	–		
2. Disclosure	45	0.44	–	
3. Positive aspects	45	0.14	0.23	–
**Social satisfaction**				
5. Friendship	45	0.50**	0.15	−0.02
6. Loneliness	45	0.52**	0.30*	0.30*
7. Rejection	45	0.56**	0.40**	0.20
8. Hostility	45	0.59**	0.33*	0.21
10. Self-esteem	45	−0.14	−0.33*	−0.44**
11. Social adaptive functioning	44	−0.11	−0.27	−0.40**
12. Self-efficacy	45	−0.43**	−0.37*	−0.20

*p < 0.05 level (2-tailed). **p < 0.01 level (2-tailed).

Regarding variables assessing social functioning, all four social satisfaction subdomain scores were significantly associated with higher discriminative stigma (all *p*’s < 0.01). Similarly, higher disclosure stigma was significantly associated with lower social satisfaction in the subdomains of loneliness, rejection, and hostility (all *p*’s < 0.05), but not friendship (*p* = 0.32). Positive aspects of stigma were significantly associated with the loneliness subdomain (*p* = 0.04), but not friendship, rejection, or hostility (all *p*’s > 0.05). Lower adaptive social functioning was associated with positive aspects (*p* = 0.01), such that people with lower adaptive social functioning scores reported less positive experiences with their autism diagnosis.

### 3.2. Qualitative

The authors used a phenomenological approach in alignment with the social identity theory to understand the participants’ lived experiences of personal stigma and explore how stigma related to their social identity (64). Past studies have examined the impact of stigma on autistic adults’ identity and wellbeing [e.g., (18)]; thus, a blended approach allowed for both existing and developing codes to emerge. The authors transcribed verbatim audio recordings of the interviews, then coded responses by identifying and labeling recurring concepts (65) via NVIVO 10. A codebook was developed to categorize concepts derived from participants’ responses into meaningful themes. The original version of the codebook aligned with the overarching structure of the interview. For example, this included sections aligning with general inquiry about stigma (i.e., types and sources) and then sections about each of the two social identity and social functioning domains. Code operational definitions were added and refined following consensus coding by two team members of several initial interviews. Additional codes were added throughout the coding process as relevant and the data was considered saturated after no novel themes emerged from the coded interviews. Questions about coding were reconciled through consensus conversations among team members.

### 3.3. Thematic analysis results

Four themes emerged from the data examined (1) type of experienced stigma, (2) source of stigma, (3) perceived reason for stigma, and (4) impact of stigma on multiple domains of social functioning. The terms ‘some,’ ‘most,’ and ‘all’ were used to quantify the number of participants who shared similar experiences. ‘Some’ is defined as less than or equal to half of the participants; ‘most’ is defined as more than half of the participants (i.e., 5 to 7); and ‘all’ is defined as all eight participants. Pseudonyms and non-binary pronouns (they/them/their) are used to personalize the responses and to maintain confidentiality.

#### 3.3.1. Type of stigma

All participants expressed experiencing some type of stigma. Definitions of stigma from the literature highlight that stigma is experienced in six primary modalities including, labeling, stereotyping, separation, status loss, discrimination and misuse of power (17). Autistic adults in the current sample provided examples of experienced stigma across most of these modalities (see Table 3).

**TABLE 3:** Examples of participants’ experienced stigma.

Theme	Example
**Type of stigma**	
Stereotyping	“And so there have been…plenty of people who I tell them my diagnosis and I get a ‘oh I would’ve never known’ or ‘you’re nothing like my cousin’s sister’s brother’s ex’s kid.’ And I’m like ‘cool it’s because I’m not your cousin’s sister’s brother’s ex’s kid’…a lot of times it makes me feel like…I don’t actually have autism but just that I’m not worthy of being part of like that community.” (Beatriz)
Separation	“As far as responding to my diagnosis I mean I never tell anyone about my diagnosis except for like this because I know they will not respond well no one has ever responded well.” (76) “I don’t want to be like because I’m a student with accommodations or anything, but I was just like she’s just making me feel kind of weird and it’s like you’re not treating other students like this.” (Kari)
Discrimination	“Parents got involved. Mom says she’s a nurse says, ‘Oh he’s gonna have a meltdown and you better not have that you know if y’all get married and then you have a child then you gonna take care of the child all your life because of the autism offspring.’ Dad says, ‘Oh you can’t have a uh you can’t be around him because he might not be able to have a job.”’ (Fatima) “I wasn’t allowed to talk about my disability at work which is kinda crazy because a lot of my students had disabilities themselves.” (Gabriel)
Misuse of power	“Um I was laid off in (county) for being disabled as well because I needed accommodations for my visual processing disorder and so when they needed to lay off half their staff, they can’t fire you for being disabled but when they have to lay off half their staff, then they can get away with it.” (Gabriel)
Overt bullying and abuse	“One time we were like sitting in his truck and he was like, ‘I’m sorry, but this truck is like actually autistic,’ because his truck was acting up.” (Kari) “Oh yeah, (laugh) I mean like I was picked on a lot in middle school so like then. Um my sister liked to call me freak for a while.” (Beatriz)
**Source of stigma**	
Educators	“I was… working on a problem on the board and it was taking me a while and (teacher) actually called me a ‘retard’ in front of the whole class for it.” (Ali)
Peers	“In high school…I was bullied a lot. ‘You’re different, you need to stop thinking about your future.’ This that and the other because I said during my high school years I wanted to go for a Ph.D. and people looked at me like you’re nuts. I know that was just my social peers.” (Fatima)
Family members	“(My sisters) would belittle me about it a few times. Like whenever I was doing something- whenever I’d say something, they didn’t agree with they’d just say, ‘Oh he’s insane.’ And they would just totally discredit me because of (my diagnosis) and that made me feel ashamed that I had something that people could just do that with.” (Ali) “My family… tried to convince me that… ‘you can’t be a medical doctor because your motor skills are bad.’ Well, you know I always said…’let me prove you wrong’ and I wasn’t told until after I graduated with my uh bachelor’s is that my parents both told me at graduation that ‘we thought you were gonna flunk out the first semester and you gonna be moving back home.”’ (Fatima)
Community members	“There’s a really nice lady in my choir, she would tell me things like- but you have Asperger’s not autism so you’re safe.” (Hanna) “Yeah when (ADA Coordinator) told me that I took her literally and I took her out of context and walked out on me, I feel very ashamed (of my diagnosis).” (Hanna)
**Consequences of stigma**	
Self-esteem	“I pretty systematically get rejected whenever I ask someone out and I don’t know how much of that is autism and how much of that is other things. But yeah. That always makes me lose confidence in myself.” (Ali) “It also made me feel really sad because I mean… It really hurt my self-confidence because I mean you’re supposed to you know try to earn the respect of your teachers through your work, and they feel like I was just totally unable to do that.” (Ali)
Social self-efficacy	“I realize that when other people respond to my autism diagnosis the wrong way, I usually spend a lot of time and effort in educating them ‘laugh’ Um, I don’t think I stopped anything, I think I become more committed to making them understand that they can’t say those things.” (Hanna) “She (social skills tutor) you know did kind of like have a conversation with me and was like look like you do want to be careful with like who you share your diagnosis with in college um because people like do have biases and they do have stereotypes and you know you’re going into a competitive field and you don’t want that to be the first thing that people know about you. You want them to like make their own um opinions about you. So, I’ve definitely like been more hesitant to like share my diagnosis and I don’t think that’s something where I’ve been like ashamed of it I’ve just like I’m aware of the realities of the world and like not everyone like knows you know what autism is or what it means.” (Beatriz)
Social satisfaction	“I often feel left out and alone being a grad student with autism.” (Hanna) “I mean, people can tell that I don’t act normal and I think that I’m a pretty easy target. So people just in general weren’t super nice to me or like very encouraging… I mean it kind of just drove like a further wedge between me and all these other people like, even though I was on the team, I never really felt like I was a part of the team” (Kari)
Adaptive socialization	“…the student alliance meeting. Um I only went to one of them at the beginning of the semester because it was kind of a social thing and I got uncomfortable with it, and I’ve been like too nervous to put myself back in that situation and go back there.” (18002)

##### 3.3.1.1. Stereotyping

Some participants shared experiences of others relying on stereotypes to make general assumptions about autism. For example, Kari explained a time they experienced stigma while having dinner with their ex-partner’s family: “Umm his stepsister was talking about her ex-boyfriend or something and she was like… ‘He had Asperger’s like, that’s why he was kind of weird,’ and then his siblings started joking about it.” Beatriz also explained how others minimized their autism because it did not align with other autistic exemplars they held (see Table 3).

##### 3.3.1.2. Separation

When asked to describe a time participants were treated unfairly, all indicated experiences of separation or exclusion from the neurotypical society because of their behavior and/or autism diagnosis. Some individuals reported being made to feel as though they did not fit in with the neurotypical society. For example, Diya shared a time when their classmates were talking about how one of their parents work with people on the autism spectrum, explaining how “they were really talking about them as (if they were) other people.”

##### 3.3.1.3. Discrimination

Some participants reported experiences of discrimination. Diya shared about an instance “at a camp that was meant for autistic people” when they felt discriminated against by camp staff: “They isolated me in the nurse’s office and told me that I was using my disability as an excuse and I was trying to just get attention by hurting myself and it honestly made me feel worse.” This form of stigma made Diya feel as if they was not seen as a person, and that they “were just looking at (them) because of (their). disability.”

##### 3.3.1.4. Misuse of power

Gabriel shared a more intense situation in which a teacher from their daughter’s school got overly involved in the child’s care because the teacher did not believe Gabriel and their partner could “protect” their daughter because they were autistic, or “disabled” as described by the teacher. Other examples discussed in more detail below involve the refusal to provide legitimate educational accommodations.

##### 3.3.1.5. Overt bullying and abuse

In addition to these types of less overt aggressions, most participants in this sample also experienced more overt types of bullying and abuse, including both physical and verbal bullying/verbal abuse. Eric explained how their “hyper fixations” imposed on their conversations with others. They knew others would make “sly comments” about this which resulted in them wanting to “shut up and not talk to people and kinda be by myself.” One participant experienced stigma in a physical manner. Ali explained: “I’d get beat up because people didn’t- I mean- people hated me there in middle school and I think a lot of that just comes down to the fact that I was different, and they didn’t understand that.” Other examples shared by participants regard instances of verbal bullying, such as name calling and using “autistic” in a colloquial manner to refer to something defective (see Table 3).

#### 3.3.2. Source of stigma

Overall, participants experienced stigma from nine different sources, including family members, peers, significant others, healthcare professionals, educators, employers, camp counselors, acquaintances, and strangers.

##### 3.3.2.1. Educators

Most participants experienced stigma from educators and this was the most prevalent source among all reported. The type of stigma experienced by educators ranged from singling students out because of their autism diagnosis or observed symptoms, to minimizing the need for legally assigned accommodations. For example, Ali shared an instance in which an educator infringed upon the use of extra time:

“Um and then there was one time sophomore year where I had like an accommodation to be able to stand in the back of the room if I just needed to like fidget or whatever and this one teacher called me out and in front of the class and he was in a pissy mood that day and just made me sit… it was just like I don’t know kind of made me insecure (and) I know what works for myself why won’t you let me. I clearly like wasn’t distracting anyone.”

Although many of the participants described experiencing stigma in high school and in their earlier developmental years, most of the participants reported still experiencing stigma in postsecondary settings as well. Hanna’s experienced stigma from their research supervisor highlighted the lack of knowledge about autism even in higher education: “He still has a lot of like stigma that are not promotive to our relationship, such as um he doesn’t understand how much variability there is among all the autistic people.”

##### 3.3.2.2. Peers

Most participants also experienced stigma from the peers at school. Stigma mainly came from acquaintances or classmates, including accounts of demeaning comments or physical bullying. One participant described experiencing stigma from a significant other after disclosing their autism diagnosis. Kari explained, “When I, you know, disclosed to him about it, you know, right after we’d started dating, he like thought that I was joking.” Kari further explained that their significant other would say things that implied that they couldn’t care for themselves because of their autism diagnosis.

##### 3.3.2.3. Family members

Family dynamics varied across participants. Unfortunately, most participants described negative relationships with different family members, while some even explained experiencing stigma from their family. Eric shared that their parents would refer to their diagnosis in a “derogatory tone:” “They’ll say something like, ‘you know well I guess it’s your duh, duh, duh diagnosis acting up today.”

##### 3.3.2.4. Community members

There were also accounts made by some participants of experienced stigma in the form of discrimination and misuse of power by community members, such as healthcare providers and employers. Gabriel explained how they were laid off from a job because of their request for accommodations:

“The same employers that laid me off um for being disabled. They said it was because I had requested accommodations for being disabled. They didn’t say it was for being disabled, they said it was for requesting accommodations and I shouldn’t have requested accommodations. And um yeah that made me feel kind of ashamed.”

Additionally, some participants experienced stigma from acquaintances and strangers. Eric was a victim of stigma when playing Dungeons and Dragons, an online video game and someone used the term “autistic” colloquially to indicate something negative.

“Like a month ago, um I’m in this group chat for dungeons and dragons and I only know like one person there and he invited me in, but you know I guess it’s like the internet thing to say oh no it’s autistic. And I’m like, ‘dude that’s—that’s not cool I have autism.”’

#### 3.3.3. Consequences of stigma

##### 3.3.3.1. Impact of stigma on self-esteem

All participants declared their social self-esteem was negatively impacted by experiences of stigma. Stereotypes about autism not only alter others’ understanding about autism, but it seemed that stereotypes also affected participants’ perceptions of themselves. Diya explained how a camp counselor’s negative views of their abilities to be independent impacted their self-esteem in the long-term, as they were questioning their ability to move away for college:

“I just couldn’t do anything I guess um like I was- like the stereotypes of like autistic people were kind of playing through my head like I’m never going to be able to leave my parents I’m always gonna be stuck here um I can’t do college because it will be too overwhelming, and even though I knew all of those were lies like I was just really depressed and overwhelmed.”

Kari’s social self-esteem was also impacted by experienced stigma. They explained how their significant other’s negative perceptions of their abilities made them “feel like (they were) like less than a person.”

##### 3.3.3.2. Impact of stigma on social self-efficacy

Participants reported variable self-efficacy in a range of situations requiring socialization, such as in the classroom, at a job, or in relationships. Most participants reported that experienced stigma had a negative impact on their self-efficacy in social situations. Ali explained how they tended to second guess or analyzed social situations after they occured. For example, when they “say something other people will laugh at and then a little bit later I’ll start thinking about how they’re probably laughing at me and not with me.”

##### 3.3.3.3. Impact of stigma on social satisfaction

In general, most of the participants reported a mix of both social satisfaction and dissatisfaction depending on interactions with others. Social dissatisfaction was related to experienced stigma for most participants. For Kari, this decrease in social satisfaction was the result of discrimination and isolation from their team members:

“I mean, people can tell that I don’t act normal and I think that I’m a pretty easy target. So people just in general weren’t super nice to me or like very encouraging… I mean it kind of just drove like a further wedge between me and all these other people like, even though I was on the team, I never really felt like I was a part of the team”

##### 3.3.3.4. Impact of stigma on adaptive socialization

Many individuals indicated that lower adaptive social skills or autism symptoms contributed to an increase in stigmatization. For example, Fatima shared: “I’m just like not sure how to keep up with the conversation and butt in the conversation to make myself relevant and sometimes I end up feeling left out a lot.” Additionally, some participants also reported that their other characteristics of autism or autism diagnosis were reasons why they experienced stigma. For example, Ali explained how they’re “kinda like off in (their) own world sometimes” and can “sometimes…come across weirdly.” Also, Fatima’s parents discouraged them from becoming a “medical doctor because (their) motor skills are bad.”

## 4. Discussion

This study provides a mixed method examination from the perspective of autistic adults on experienced stigma and how it relates to a range of social outcomes. Results from this study replicate previous research demonstrating that autistic individuals experience high rates of stigma (5, 10). Qualitative data helped to reveal that these individuals experienced a myriad of different types of stigma that come from a wide range of sources. This information documents that in spite of the significant strides made by the neurodiversity movement toward reducing stigma (3), most autistic adults in this sample report still experiencing stigmatizing interactions in recent years in employment, postsecondary education, and from peers. This study highlights the need for more specific trainings in workplace and educational settings to increase awareness of the different types of implicit and explicit stigma people often engage in and continue to grow alignment with a neurodiversity mindset to shift the culture toward more acceptance.

With regard to the negative consequences of stigma, the current study also expands the literature [e.g., (5, 35)] by specifically examining the relation between experienced stigma and components of the social identity theory thought to contribute to an internalization of stigma: self-efficacy and self-esteem. Quantitative results revealed individuals reporting higher amounts of experienced stigma had significantly lower self-efficacy and self-esteem. Delving into these associations in more detail, interviewees revealed that the misconceptions held by others about autism often resulted in them feeling more negative about themselves or “less than a person.” Stigmatizing experiences resulted in low expressed self-efficacy in social situations and employment seeking. While not explicitly examined in this study, these findings help to elucidate how experienced stigma transitions to self-stigma and ultimately, mental health concerns among autistic individuals (5, 10, 23, 24, 28–30). Because systemic acceptance continues to spread at a pace that might be insufficient to help autistic adults that might have already endured a great deal of stigma, understanding more about the potential mechanism between experienced stigma and later mental health consequences helps to understand that more resources should focus at present on helping to bolster self-esteem and self-efficacy among neurodiverse populations.

This study also documented a link between experienced stigma and metrics of social functioning, such as social satisfaction and adaptive social skills. Although all of the research questions examining the true impact of stigma would benefit from longitudinal studies, the social success variables are most difficult to interpret with a cross-sectional, correlational design because it is likely that there is a cyclic pattern. Previous research has shown that social success is likely both a predictor of experienced stigma and an outcome [i.e., (4, 18, 21, 33)]. Qualitative data from this study reveal a similar pattern in that participants report that the different social abilities they possess contributed to greater experiences of stigma and that increased stigma led to less overall social satisfaction. This confirms the importance on conducting more longitudinal research in this area to better understand how stigma impacts quality of life.

The positive aspects subscale of the stigma measure did not align with the discrimination and disclosure subscales in terms of a relation with self-esteem and self-efficacy. As a reminder, on this subscale a higher score indicated that an individual had less positive experiences attributable to their autism diagnosis, which is an important, but much different, aspect of stigma compared to the others measuring more overtly negative experiences. Although we still anticipated that this metric would significantly relate to the social identity indices, it is likely that other participant characteristics not assessed in this study impacted this relation. For example, research shows that autistic adults that align with a neurodiversity movement mindset (3, 66) and those with a stronger affiliation to their autistic identity (67) have a more positive self-esteem and a positive social identity. Future research would benefit from including other protective and predictive factors in the model to determine among what groups and in what context stigma most likely leads to internalization and subsequent mental health concerns.

### 4.1. Limitations and future directions

There were several limitations of this study that are important to note. One key limitation is that primarily autistic individuals with higher cognitive abilities participated, such as those attending college or maintaining full-time employment. This limits the ability to generalize findings to the entire autism population, including those with lower intellectual functioning and proliferates the issue that autistic individuals with higher support needs are underrepresented in the autism literature (68). Because individuals with intellectual disabilities also face stigma in society (69), future research should also recruit participants with autism and co-occurring intellectual disability to understand if they have unique stigma experiences.

As participants were recruited through various methods to complete an online survey about autism and experienced stigma, selection bias could have influenced results (70). Perhaps only those who felt as if they experienced stigma participated in the study, leaving out others with different experiences. The nature of phenomenological research also limits the generalizability of findings (71). Although the qualitative interviews served as a follow-up to better understand and apply deeper meaning to the quantitative results (45), participants’ lived experiences are unique to the individual and cannot appropriately explain all autistic adults’ experiences.

Another limitation to the study is the lack of racial and ethnic diversity in the qualitative sample and a failure to document the demographic composition of the quantitative sample, which prohibited researchers from controlling for demographic factors in the analyses. The fact that mainly white, non-Hispanic autistic individuals participated in the qualitative interviews limits the understanding of intersectionality of identities. For example, Black autistic individuals experience racial discrimination from society, in addition to ableism from their community [see (72) for a review]. As the majority of the research has focused on the relation between cultural and affiliate stigma [e.g., (29)], future research should specifically explore stigma among autistic adults with a more diverse intersection of identities to better understand if different groups have unique stigma experiences.

Finally, there were limitations in the sample size and reliability analysis. Specifically, the small sample size of the quantitative study limited the ability to perform more complex quantitative analyses. Future research should employ methods that allow for an examination of social identity variables as mediators between stigma and reported symptoms of psychopathology and those that examine more cyclical patterns in how social ability might serve as a potential predictor and outcome of stigma. Most importantly longitudinal research is needed to really examine whether the experienced stigma over time is a causal factor in contributing to lower social identity and social success. Furthermore, the lower Cronbach’s alpha that was calculated on the Social Self-Efficacy subscale limits our confidence that the subscale accurately measures social self-efficacy; however, a 0.6 alpha is considered moderately acceptable or satisfactory in some literature for the use of psychology measurement in research [see (55) for a review].

## 5. Conclusion

The pervasive and prolonged nature of stigma experienced by autistic individuals indicates that efforts to impart change continue to be insufficient. As rates of autism continue to rise and more supports are put into place, more autistic adults are predicted to enter post-secondary education or professional settings (73) thus, we need approaches to reduce stigma in childhood and adult context. By examining how stigma relates both social outcomes and core features of one’s social identity we can continue to alert the public to the importance of engaging in stigma reduction efforts in educational institutions or workplaces and to develop and implement more appropriate support structures for autistic students or employees to mitigate these negative experiences. This research helps to underscore the importance of continued efforts to help improve societal attitudes about autism through great acceptance to reduce harmful stigma and to help mitigate the subsequent negative social consequences (7). Also, given that many autistic adults have already encountered stigma, understanding the extent of the consequences and how we might help to ameliorate these is essential.

## Data availability statement

The raw data supporting the conclusions of this article will be made available by the authors, without undue reservation.

## Ethics statement

The studies involving humans were approved by the University of Georgia Institutional Review Board. The studies were conducted in accordance with the local legislation and institutional requirements. The participants provided their written informed consent to participate in this study.

## Author contributions

AM and AH contributed to the conception and design of the study. AH supervised AM during the development of the codebook and interview protocol. AM collected with majority of the quantitative and qualitative data, with help from KB, GT, and AH. KB, GT, and AH analyzed the quantitative data. KB and AH identified themes in the qualitative data. All authors wrote sections of the manuscript, contributed to manuscript revisions, and approved the submitted version.

## Author disclosure

There is clear indication that autistic adults prefer identify-first language [e.g., (74)]. Despite the disagreement among professionals about the use of identify-first versus person-first language, we opted to use identify-first language (i.e., autistic individuals) to align with autistic adults’ preference as the participants in this study. The use of this language is consistent with APA guidance https://apastyle.apa.org/style-grammar-guidelines/bias-free-language/disability#:∼:text=Avoid%20language%20that%20uses%20pictorial,AIDS%E2%80%9D%20or%20%E2%80%9Cperson%20with%20a.
